# Inhibitory effect of allicin and garlic extracts on growth of cultured hyphae 

**Published:** 2014-03

**Authors:** Farzad Aala, Umi Kalsom Yusuf, Rosimah Nulit, Sassan Rezaie

**Affiliations:** 1 Department of Medical Mycology & Parasitology, School of Medicine, Kurdistan University of Medical Sciences, Sanandaj, Kurdistan, Iran; 2 Department of Biology, Faculty of Science, University Putra Malaysia, Selangor, Malaysia; 3 Department of Medical Mycology & Parasitology, School of Public Health, Tehran University of Medical Sciences, Tehran, Iran

**Keywords:** Allicin, Dermatophytosis, Electron microscopy, Garlic (*allium sativum*) extract, * Trichophytonrubrum*

## Abstract

***Objective(s):*** *Trichophyton rubrum* (*T. rubrum*) is one of the most common dermatophytes worldwide. This fungus invaded skin appendages of humans and animals. Recently, resistance to antifungal drugs as well as appearance of side effects due to indication of these kinds of antibiotics has been reported. Besides, using some plant extracts have been indicated in herbal medicine as an alternative treatment of these fungal infections. The aim of this study was to investigate the effects of Garlic (*Allium sativum*) and pure allicin on the growth of hypha in *T. rubrum* using Electron miscroscopy.

***Materials and Methods:*** This study was carried out to observe the morphological changes of *T. rubrum* treated with allicin as well as aqueous garlic extract using scanning electron microscopy (SEM) and transmission electron microscopy (TEM).

***Results:*** SEM surveys, showed that hypha treated with allicin has rough and granular like surface, abnormal and irregularly-shape. However, hypha treated with garlic extract had rough and fluffy surface and also irregularly-shape. TEM studies also found that hypha treated with allicin displays disintegration of cytoplasm, breaking down in cell membrane and the cell wall, and collapsing of hypha, meanwhile hypha treated with garlic extract exhibiting degradation and dissolution of cytoplasm components, demolition of cell wall and cell membrane, and hypha appeared to break.

***Conclusion:*** The present study revealed that pure allicin (6.25 µg/ml and 12.5 µg/ml) is more efficient in inhibition of the growth in hyphal cells compare to the garlic extract (2 mg/ml and 4 mg/ml) and they could be used as alternatives in treatment of dermatophytosis.

## Introduction

Dermatophytes are a group of keratinophylic filamentous fungi infecting the skin and skin appendages of humans and animals. One of the most frequently isolated dermatophytes is *Trichophyton rubrum*(1-2). Antifungal agents such as imidazole and triazole drugs have been used in the treatment of various fungal infections. However, resistance to these drugs and the appearance of their side effects as well as their toxicity due to the administration of these drugs have been reported(3-5). As an alternative, plant extracts in herbal medicine have been used in the treatment of dermatophytosis. One of the plant extracts which has been used in this way is garlic (*Allium sativum*). This plant has been known to have antimicrobial, anti-inflammatory, anti-thrombotic and antitumor activities. Previous *in vitro* studies indicated the effects of garlic extract on the inhibition of the growth in a large number of yeasts including *Candida* spp., some fungi such as *Coccidioides immitis*(6-9), and also dermato-phytic fungi *T. rubrum, T. mentagrophytes, T. verrucosum, Microsporum canis *and* Epidermophyton flocossum*(10). A sulphur-containing compound in garlic, known as di-allyl thiosulfinate (allicin), is the active component in inhibition of the growth of fungi and bacteria (11). Fresh aqueous extract of garlic showed antifungal activity specifically against some *Aspergillus *spp. including *A.* *fumigates, A.* *terreus, A. nidulans, and A. niger* (12).The inhibitory effects of fresh aqueous extract of garlic against *Aspergillus *spp. were revealed in different concentrations. The inhibitory effects of allicin against *Trichophyton* spp are more pronounced than those of the essential oils derived from other plants (5). Although many investigations showed inhibitory effects of the plant extract on fungal or bacterial growth, very few studies have explained the its mechanism against fungi and bacteria. Electron microscopy has been employed to understand the mechanism of action of the plant extract against fungi and bacteria at cellular level. The morphological abnormalities in hyphal compartments of *T. rubrum* and *T. mentagrophytes* have been previously shown using SEM and TEM after treatment of these dermatophytes with *Allium sativum* extract (13).In addition, other investigation has revealed the effects of four major constituents of oil essences on the morphology and ultrastructure of hyphae in *T. mentagrophytes* and their antifungal activities (14).In the present study, we attempted to find out the ultrastructural characteristics of *T. rubrum* in response to allicin as well as garlic aqueous extract by SEM and TEM approaches.

**Figure 1:**
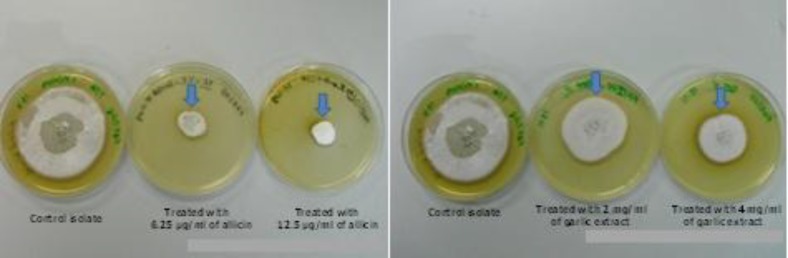
The effects of allicin and the fresh garlic-extract on the cultures of *Trichophyton rubrum*. The radial growth of *T. rubrum* colonies was reduced as compared to the control

## Materials and Methods

***Preparation of antifungal agents and*** ***T. rubrum***

Allicin (Alexis-Biochemicals Co, San Diego, USA) was dissolved in 10 mg/ml of methanol/ water/formic acid (60:40:0.1) and stored at -20°C. Garlic aqueous extract was prepared from fresh garlic based on modified method of Ghahfarokhi *et al*) (15). *T. rubrum* (ATCC 10218) was cultured on sabouraud dextrose agar (SDA) (Difco Laboratories, Detroit, Michigan, USA) and incubated at 28°C for 14 days.

***Culture conditions for the microscopic observation***

Minimal inhibitory concentration (MIC_90_) was used as low concentration treatments for each allicin or garlic extract. For attaining fungal mycelia exposed to concentrations of allicin and/or garlic extract, sabouraud dextrose broth (SDB) culture medium was used. *T. rubrum* was treated with different concentrations of allicin (6.25, 12.5 and 25.0 µg/ml) and garlic extracts (0.25, 0.5, 1.0, 2.0, 4.0 and 8.0 mg/ml). Minimal fungicidal concentration (MFC) was used as the highest concentration in treatments of allicin and garlic extract based on the previously described method of Park *et al *(14). Briefly, three culture plugs of *T. rubrum* were inoculated with 100 ml liquid culture media (SDB) and cultured in shaking incubator for 2 days at 28°C. Allicin (12.5 µg/ml) and garlic extract (4 mg/ml) were added to medium and incubated for 3 more days at 28°C. The mycelia mass was then harvested to perform electron microscopy investigations.

***Specimen preparation for SEM and TEM***

Samples for SEM observation were prepared according to Park *et al *and Iwasawa *et al* methods with some modifications (14-16).The isolates were harvested after being treated with allicin or garlic extract. A specimen of each isolate was fixed with 4% (v/v) glutaraldehyde at 4°C, overnight. The fixed mycelia were then washed three times with 0.1 M Sodium sodium cacodylate buffer (pH 7.4). The samples were postfixed with 1% (v/v) osmium tetroxide at 4°C for 2 hr. The postfixed mycelia were then washed again three times for 10 min with 0.1 M Sodium sodium cacodylate buffer. The postfixed samples were then dehydrated in a graded ethanol series (from 30% to 90%), each for 10 min, and then dehydrated in ethanol 100% three times for 15 min. The dehydrated specimens were dried with liquid carbon dioxide in a critical point drier (BAL- TEC, CPD 030, Germany) for 30 min. The dried samples were covered with gold by applying a sputter coater (BAL- TEC, SCD 005, Germany). Finally, the specimens were visualized by a scanning electron microscope (Philips XL30- ESTM, USA) at 20 kV.

Samples for TEM observation were prepared according to the methods described by Park *et al* and Iwasawa *et al* with some modifications (14-16). The isolates were selectively collected after being treated with allicin or garlic extract. A specimen for each isolate was fixed with 4% (v/v) glutaraldehyde at 4°C, overnight. The fixed mycelia were washed three times for 10 min with 0.1 M sodium cacodylate buffer (pH 7.4). Samples were postfixed with 1% (v/v) osmium tetroxide at 4°C for 2 hr. The postfixed mycelia were washed again three times (each time for 10 min) with 0.1 M sodium cacodylate buffer (pH 7.4). Then, the postfixed samples were dehydrated in a graded acetone series (from 35% to 95%), each for 10 min. In the next step, the samples were dehydrated in acetone 100% three times for 15 min. 

**Figure 2:**
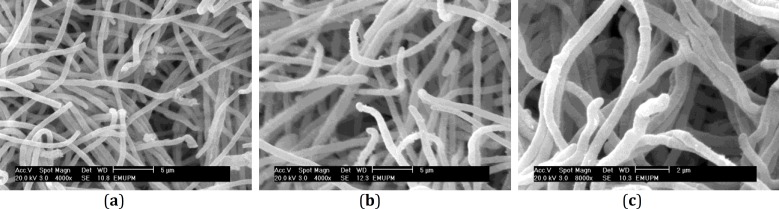
Scanning electron micrographs of *Trichophyton. rubrum*** a.** typical hypha was recognized by smooth like surface** b.** *T. rubrum* treated with allicin (12.5 µg/ml) showing hyphae with rough and granular like surface** c.** *T. rubrum* treated with garlic extract (4 mg/ml) showing hyphae with rough and fluffy surface

The dehydrated samples were infiltrated twice with acetone and resin combination for 1 hr and 2 hr correspondingly. The samples were then kept in 100% resin, overnight. Next, samples were embedded in Spurr’s resin and polymerized in oven (Memmert UIS, Western Germany) at 60°C for 24-48 hr. Samples were cut into ultra-thin sections by ultramicrotome (Leica UCT, Austria). Prepared sections were stained with uranyl acetate for 10 min followed by lead citrate for 10 min. A transmission electron microscope (Philips EM 400- HMG, Holland) at 80 kV was used to perform the observations of the stained sections. Untreated isolates were used as controls.

## Results

The radial growth of *T. rubrum* cultured on SDA medium and treated with 6.25 µg/ml, 12.5 µg/ml of allicin and 2 mg/ml, 4 mg/ ml of garlic extract was decreased in comparison with the control. In addition, the inhibition of *T. rubrum* growth by allicin was found to be more as compared with the garlic aqueousextract (Figure 1).

Scanning electron micrographs of *T. rubrum* are shown in Figure 2. The comparison between normal and treated (allicin and garlic extract) hypae showed smooth walls in *T. rubrum* normal hyphae in SEM observation. However, hyphae which were treated with allicin (12.5 µg/ml) were found with rough and granular like surface. Also, SEM observation revealed rough and fluffy surface for hyphae which were treated with 4 mg/ ml of garlic extract (Figure 2).

**Figure 3:**
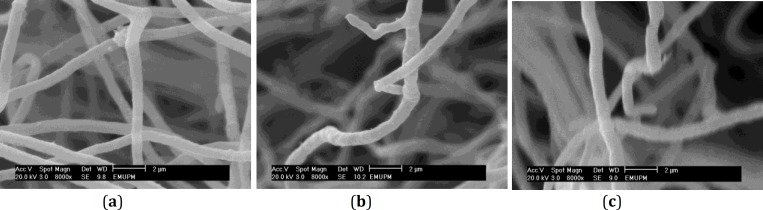
Scanning electron micrographs of *Trichophyton rubrum*. **a.** Control hyphae without any treatment which demonstrates clear straight shapes. **b.** *T. rubrum* treated with allicin (12.5 µg/ml) which demonstrates hypha with abnormal and irregular shapes. **c.** *T. rubrum* treated with garlic extract (4 mg/ml) which demonstrates hyphae with irregular shapes

Furthermore, micrograph of normal hyphae illustrated a straight-shape. However, micrographs showed that hyphae treated with allicin (12.5 µg/ml) had abnormal and irregular shape. Besides, hyphae treated with garlic extract (4 mg/ml) appeared to have irregular shape (Figure 3).

**Figure 4:**
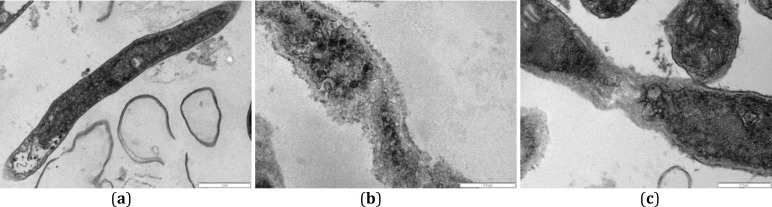
TEM studies of a longitudinal section of *Trichophyton rubrum*. **a.** Normal untreated hypha showing typical cell wall, cell membrane and organelles. **b**. Hyphae treated with allicin (12.5 µg/ml) showing disintegration and deterioration of cytoplasm, break down of the cell membrane and cell wall, and collapse of hyphae.** c**. Hyphae treated with garlic extract (4 mg/ml) indicating degradation and dissolution of cytoplasm components, demolition of the cell wall and cell membrane, and hyphae appeared to break. Scale bar=0.5 µm

TEM results of the treatment of *T. rubrum* isolates with allicin and garlic extract are shown in Figure 4. Micrograph exhibits normal/untreated hypha with typical cell wall, cell membrane and organelles. In contrast, hyphae treated with allicin (12.5 µg/ml) showed disintegration and deteriora-tion of cytoplasm parts, as well as breakdown of the cell membrane and cell wall, and collapse hyphae. Besides, hyphae treated with garlic extract (4 mg/ml) exhibit degradation and dissolution of cytoplasm components in addition to destruction of cell wall and cell membrane. Hyphae appeared to break in this condition (Figure 4).

## Discussion

This investigation was conducted to reveal the interaction of allicin and garlic extract and hyphae in the dermatophyte pathogen, *T. rubrum*. Generally, allicin was indicated to have more effective inhibitory affects on the growth of fungal cells as compared to garlic extract. SEM studies showed normal hyphae with smooth walls, but hyphae treated with allicin (12.5 mg/ml) exhibited rough and granular like surface, and hyphae treated with garlic extract (4 mg/ml) displayed rough and fluffy surface. Yoshida *et al* (17) found that hyphae treated with 20 µg/ml of ajoene, exhibited flat ribbon like structure or surface demolition. Park *et al *(14) described hyphal abnormality such as shrinkage in *T. mentagrophytes* when treated with 0.09 mg/ml of citral.

Also, our results show that normal hyphae have a straight shape. However, the morphology of hyphae become abnormal and irregularly shaped when treated with allicin and they have an irregular shape when treated with garlic extract. These results are similar with the results of Romagnoli *et al* (18) who described that hyphae of *T. rubrum* with GI-P treatment (50 µg/ml) had enlarged hyphal tips. Based on the SEM micrographs, allicin and garlic extract caused morphological changes in *T. rubrum*. After the treatment of *T. rubrum* with allicin, rough and granular like surface, and abnormal and irregular shape were observed. However, after the treatment of *T. rubrum* with garlic extract, rough and fluffy surface and also irregular shape were observed. 

According to TEM study, a normal untreated hypha was described by typical cell wall, cell membrane and organelles. Treated cultures with allicin (12.5 µg/ml) exhibited disintegration and deterioration of cytoplasm parts, as well as breakdown of the cell membrane and cell wall, with collapse of hyphae. Besides, treated cultures with garlic extract (4 mg/ml) displayed degradation and dissolution of cytoplasm components in addition to destruction of cell wall and cell membrane, as well as break of hypha. These results were in accordance those reported by Park* et al *(14) in which untreated *T. mentagrophytes* possessed normal cell wall, mitochondrion and vacuole, but hyphae exposed to 0.2 mg/ml citral showed local thickening and discontinuity of plasma membrane. Our results are also similar with those published by Ghahfarokhi* et al* (13) reporting detachment of the exterior membrane, and also separation from the cell wall and degradation of cytoplasm content in treated cultures of *T. rubrum*. Studies by Park *et al* (14) also revealed separation of plasma membrane from the cell wall when hyphae were exposed to 0.09 mg/ml citral.

In general, disintegration of cytoplasm, breakdown of the cell membrane and cell wall, and collapse of hyphae were observed when *T. rubrum* treated with allicin and garlic extract. Therefore, it can be concluded that these two compounds are effective for cellular modification of hyphaa. However, allicin was more effective in making cellular changes in hyphae as compared with garlic extract.

Result from SEM and TEM indicated morphological and cellular modifications of *T. rubrum *caused by allicin and garlic extract. Although both of these compounds had a lot of significant effects on *T. rubrum,* but based on our results allicin was more effective in comparision to garlic extract which is probably due to the differences of the susceptibility (MICs) of* T. rubrum* towards each of these two compounds. The findings here are in line with those reported by Park *et al* (14). On the other hand, the morphological modifications of hyphae might have resulted in the demolition of organelles in the cytoplasm, which was obsereved in TEM micrographs. We could clearly observe the devastation and dissolution of cytoplasm components in hyphae exposed to allicin and garlic extract. On the basis of our results which are also in agreement with those reported by Park *et* *al* (14), morphological variations resulted in the disintegration of cytoplasmic compartments as revealed in TEM observations.

## Conclusion

In this study, we found that allicin and garlic extract possess antifungal activity which inhibits the hyphal growth of *T. rubrum*. The morphological and cellular modifications of *T. rubrum* treated with allicin and garlic extract observed by SEM and TEM demonstrated the antifungal activity of these two agents. Allicin and garlic extract as natural products can be used for medical purposes due to their antifungal activity and availability.

## References

[1] Santos DA, Hamdan JS (2005). Evaluation of broth microdilution antifungal susceptibility testing conditions for Trichophyton rubrum. J Clin Microbiol..

[2] Barros ME, Santos DA, Hamdan JS (2007). Evaluation of susceptibility of Trichophyton mentagrophytes and Trichophyton rubrum clinical isolates to antifungal drugs using a modified CLSI microdilution method (M38-A). J Med Microbiology..

[3] Al-Mohsen I, Hughes WT (1998). Systemic antifungal therapy: past, present and future. Ann Saudi Med..

[4] Odds FC, Brown AJP, Gow NAR (2003). Antifungal agents: mechanisms of action. Trends Microbiol.

[5] Pyun M, Shin S (2006). Antifungal effects of the volatile oils from Allium plants against Trichophyton species and synergism of the oils with ketoconazole. Phytomedicine..

[6] Ismaiel AA, Rabie GH, Kenawey SE, Abd El-Aal MA (2012). Efficacy of aqueous garlic extract on growth, aflatoxin B1 production, and cyto-morphological aberrations of Aspergillus flavus, causing human ophthalmic infection: topical treatment of A. flavus keratitis. Braz J Microbiol..

[7] Karuppiah P, Rajaram S (2012). Antibacterial effect of Allium sativum cloves and Zingiber officinale rhizomes against multiple-drug resistant clinical pathogens. Asian Pac J Trop Biomed..

[8] Brilhante RS, de Lima RA, Caetano EP, Leite JJ, Castelo-Branco Dde S, Ribeiro JF, Bandeira Tde J, Cordeiro Rde A, Monteiro AJ, Sidrim JJ, Rocha MF (2013). Effect of farnesol on growth, ergosterol biosynthesis, and cell permeability in Coccidioides posadasii. Antimicrob Agents Chemother..

[9] Ghannoun MA (1988). Studies on the anticandidal mode of action of Allium sativum. J Gen Microbiol..

[10] Aala F, Yusuf UK, Khodavandi A, Jamal F (2010). In vitro antifungal activity of allicin alone and in combination with two mediations against six dermatophytic fungi. Afr J Microbiol Res..

[11] Cavillito CJ, Buck JS, Suter CM (1994). Allicin, the antibacterial principle of Allium sativum, II Determination of the chemical structure.. J Am Chem Soci..

[12] Pai ST, Platt MW (1995). Antifungal effect of Allium sativum (garlic) extracts against the Aspergillus species involved in otomycosis. Lett Appl Microbiol..

[13] Ghahfarokhi MS, Goodarzia M, Razzaghi Abyanehb M, Al-Tiraihic T, Seyedipourd G (2004). Morphological evidences for onion-induced growth inhibition of Trichophyton rubrum and Trichophyton mentagrophytes. Fitoterapia..

[14] Park MJ, Gwak KS, Yang I, Kim KW, Jeung EB, Chang JW (.2009). Effect of citral, eugenol, nerolidol and α-terpineol on the ultrastructural changes of Trichophyton mentagrophytes. Fitoterapia.

[15] Ghahfarokhi MS, Razafsha M, Allameh A, Razzaghi Abyaneh M (2003). Inhibitory effects of aqueous onion and garlic extracts on growth and keratinase activity in Trichophyton mentagrophytes. Iran Biomed J.

[16] Iwasawa A, Saito K, Mokudai T, Kohno M, Ozawa T, Niwano Y (2009). Fungicidal Action of Hydroxyl Radicals Generated by Ultrasound in Water. J Clin Biochem Nutr..

[17] Ledezma E, Apitz-Castro R (2006). Ajoene the main active compound of garlic (Allium sativum): a new antifungal agent. Rev Iberoam Micol..

[18] Romagnoli C, Mares D, Bruni A, Andreotti E, Manfrini M, Vicantini CB (2001). Antifungal activity of 5 new synthetic compund vs T rubrum and Epidermohyhton floccosum.. Mycopathologia..

